# Chemoport Insertion by Interventional Radiology: Real-Life Experience in a Latin American Setting

**DOI:** 10.7759/cureus.101879

**Published:** 2026-01-19

**Authors:** Oscar F Vargas, Juliana Salcedo Mesa, Valentina Lugo-Mesa, Mateo Barros, Laura Álvarez

**Affiliations:** 1 Interventional Radiology, Hospital Departamental de Villavicencio, Villavicencio, COL; 2 Epidemiology, Universidad de los Andes, Bogotá, COL; 3 Oncology, Fundación Santa Fé de Bogotá, Bogotá, COL

**Keywords:** chemotherapy, complications, cross-sectional study, internal jugular vein, interventional radiology, port catheter, vascular access device, venous port systems

## Abstract

Introduction

Totally implantable venous access devices are essential for long-term chemotherapy, improving patients’ quality of life. Despite advances in interventional radiology, catheter-related infections and thrombosis remain leading causes of premature device removal, particularly in resource-limited settings where procedural variability persists. This study reports real-world outcomes of an interventional radiology-guided chemoport program at a Colombian public hospital, exposing a standardized, safe implantation technique and situating results within international best practices to support evidence-based vascular access practices in oncology care.

Methods

A retrospective, descriptive, cross-sectional observational study was conducted in a regional referral center located in the Orinoquia region of Colombia to characterize the complications associated with the insertion and use of PowerPort® Implanted Port System (Becton Dickinson, Franklin Lakes, NJ) for oncologic indications. Covering the period from January 1, 2023, to June 30, 2025. Our team collected the data retrospectively from electronic medical records, including demographic, clinical, and procedural variables such as age, sex, cancer type, procedure type (insertion or removal), port site, intraoperative events, and the cause of port removal when applicable. Complications were predefined by the authors before data extraction according to relevant literature: early (≤2 weeks) and late (>2 weeks) complications. Data were analyzed using RStudio (version 2024.12.1 + 563, Posit, Boston, MA).

Results

A total of 160 patients underwent a total of 170 procedures with the interventional radiology unit: 155 (91.17%) for chemoport insertion and 15 (8.82%) for device removal. Regarding the anatomical site of insertion, 134 (78.82%) chemoports were placed in the right internal jugular vein and 21 (14%) in the left internal jugular vein. As for complications, there were no early complications recorded during the study period, and only four cases of late complications were documented, which corresponded to two cases of thrombosis, one case of discomfort, and one case of vena cava occlusion. No other complications were documented.

Conclusions

Chemoport insertion using ultrasound-guided internal jugular access with fluoroscopic confirmation is the current preferred approach. The results from our study reinforce the safety and practicality of this type of procedure, which is simple to perform by interventional radiologists. The findings support ultrasound-guided, fluoroscopy-assisted chemoport insertion as a safe approach in a real-world hospital setting. Although we did not conduct a formal safety study, which may limit its comparability due to being based only on complication counts, this study gives valuable insight into its safety. Further research into this topic is needed to evaluate its efficacy in other types of patients who require long-term intravenous therapy.

## Introduction

Central venous totally implantable access devices (TIVADs, "chemoports”) were developed to overcome the limitations of repeated peripheral cannulation in oncology, preserving venous capital and enabling safe delivery of vesicants over prolonged courses [1,2]. These devices were developed in the 1980s and allowed repeated and safe infusion of substances such as chemotherapeutic agents, parenteral nutrition, and transfusions, minimizing peripheral vein trauma and improving patients' quality of life [3].

As a consequence, the need for standardized vascular access techniques has become more pronounced, particularly in resource-limited settings. Complications, such as catheter-related infections or thrombosis, although often preventable, still constitute the leading reasons for premature device removal and treatment interruption [4]. Evidence-based management can help mitigate these risks. Structured protocols for extravasation management limit tissue injury and help preserve device function [5]. Contemporary strategies improve outcomes in catheter-related thrombosis and selective device removal when complicated by sepsis, deep vein thrombosis, or persistent dysfunction [6].

Interventional radiology has progressively reduced procedural risk through real-time vascular visualization, improving technical accuracy, and reducing complication rates. Yet implementation disparities persist across public hospitals in Latin America, where financial limitations, workforce shortages, and the absence of institutional guidelines impact procedural consistency. The variability in venous approach (subclavian or versus jugular) remains a relevant determinant of safety outcomes. Evidence suggests that continuous operator experience and standardized technique adoption protocols substantially decrease complication frequency [7,8]. Against this backdrop, our objective is to report real-world outcomes from an interventional radiology-guided chemoport program in a Colombian public hospital, situating results within international benchmarks and highlighting practical implications for oncology services operating under resource constraints.

## Materials and methods

We conducted a retrospective, descriptive, cross-sectional observational study to characterize the complications associated with the insertion and use of implantable venous access ports for chemotherapy in oncologic patients (chemoports). The study was performed at the Hospital Departamental de Villavicencio, a regional referral center in the Orinoquia region of Colombia, which has an interventional radiology unit, covering the period from January 1, 2023, to June 30, 2025. All patients aged 18 years or older who underwent insertion or removal of an implantable port for oncologic indications under angiographic guidance in the interventional radiology service were included. The step-by-step insertion technique is illustrated in the following figures. Patients were excluded if they had non-oncologic indications (such as parenteral nutrition, transfusions, prolonged antibiotic therapy, or other intravenous infusions), as well as pregnant women, procedures performed without angiographic control, and cases with incomplete medical records. 

A non-probabilistic consecutive sampling strategy was applied, including all eligible cases during the study period. Data were collected retrospectively from electronic medical records (DINÁMICA software) with demographic, clinical, and procedural variables such as age, sex, cancer type, procedure type (insertion or removal), port site, intraoperative events, early (≤2 weeks) and late (>2 weeks) complications, and the cause of port removal when applicable. Data were analyzed using RStudio (version 2024.12.1 + 563). Descriptive statistics were used to summarize data; categorical variables were expressed as frequencies and percentages, while continuous variables were expressed as means or medians. Details regarding the techniques of insertion and removal were obtained from the expertise of the radiologist of our service. The study received approval from the Ethics Committee of the Hospital Departamental de Villavicencio. 

Insertion technique

Laterality and Access Selection

The laterality of chemoport implantation depends on the side of the primary cancer and any history of lymph node dissection. In some cases, anatomic variations or lesions in the innominate vein may make the contralateral side more suitable for venous access. For example, in patients with right breast cancer, implantation via the left internal jugular vein is preferred. Likewise, when internal jugular access is not feasible, placement through the external jugular vein may be considered.

Preparation and Anesthesia

**Figure 1:**
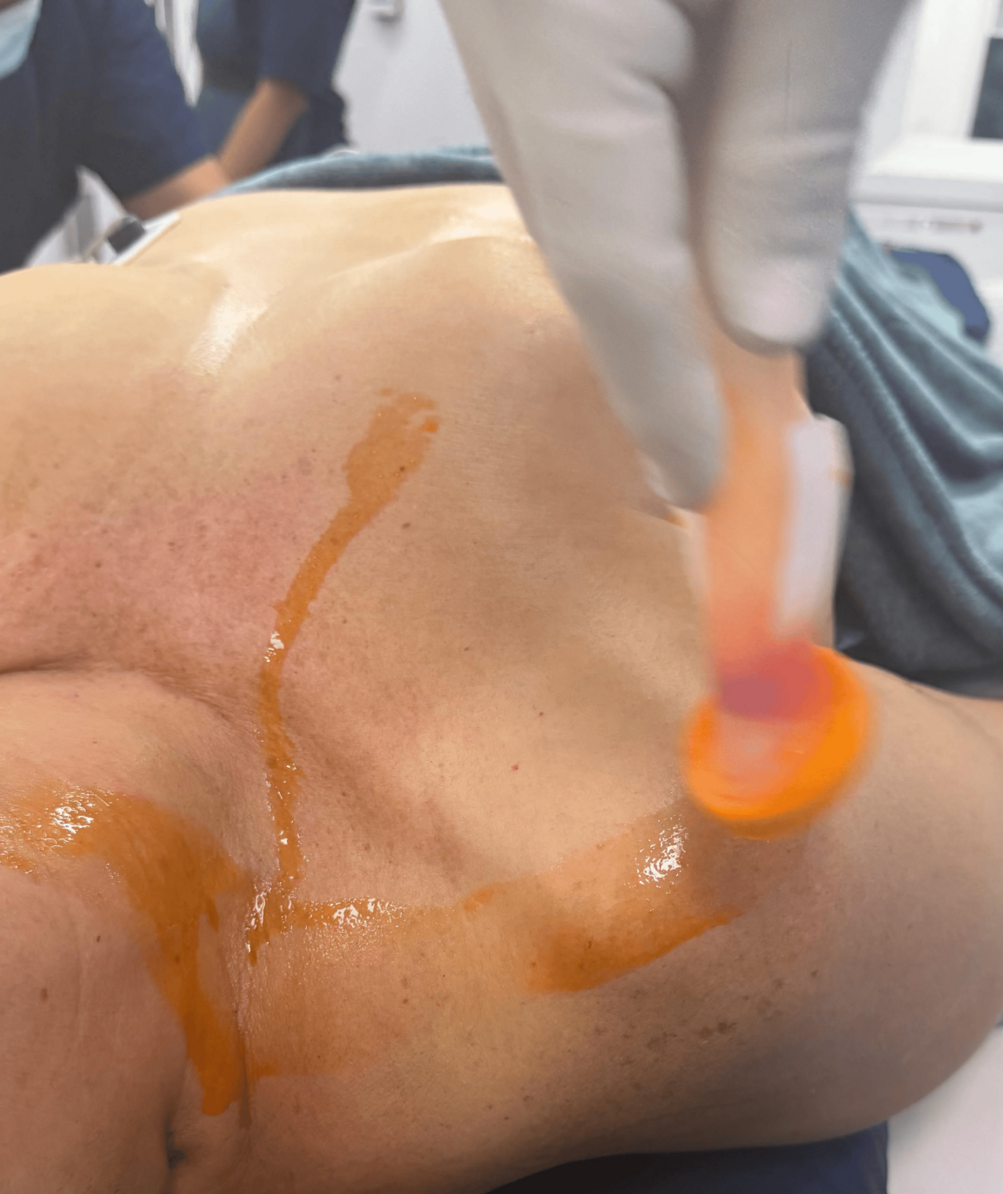
Aseptic and antiseptic skin preparation prior to the procedure Skin antisepsis was performed using a sterile solution containing 2% chlorhexidine gluconate and 70% isopropyl alcohol, applied as surgical skin preparation.

Before the procedure, informed consent was obtained. Rigorous aseptic and antiseptic skin preparation was performed using ChloraPrep with Tint (Becton Dickinson, Franklin Lakes, NJ) with 2% chlorhexidine gluconate, 70% isopropyl alcohol, and sterile solution (10.5 mL) as a surgical scrub (Figure 1). The procedure was carried out under anesthesiologist-directed sedation and, occasionally, under general anesthesia based on patient preference, with real-time color Doppler ultrasound guidance.

Venous Access

**Figure 2:**
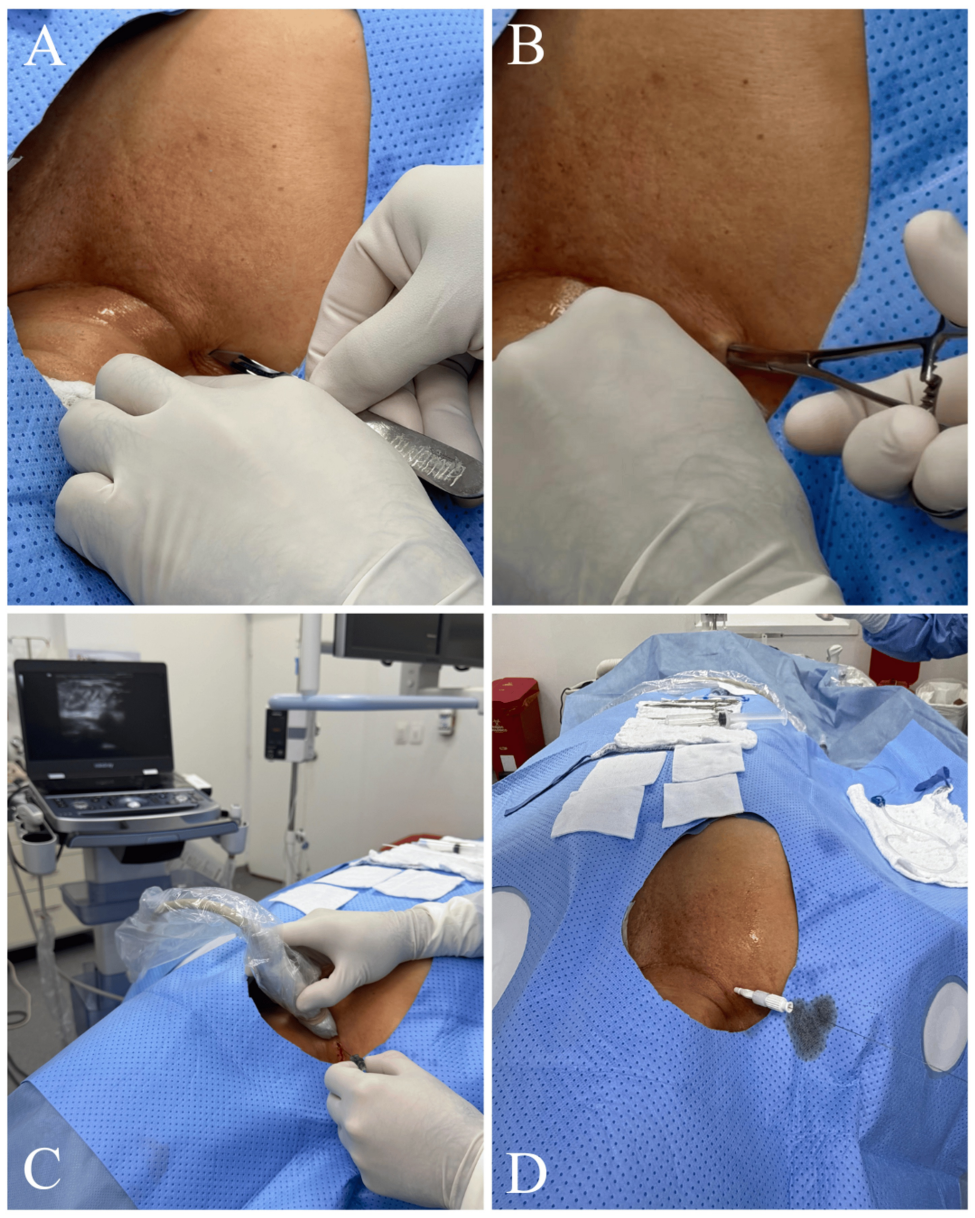
Venous access technique under ultrasound guidance (A) A 3-mm skin incision was made to facilitate access through the subcutaneous tissue. (B) Blunt dissection of the soft tissues was performed using mosquito forceps. (C, D) Under color Doppler ultrasound guidance at the supraclavicular level, venous access was obtained using a micropuncture set.

**Figure 3:**
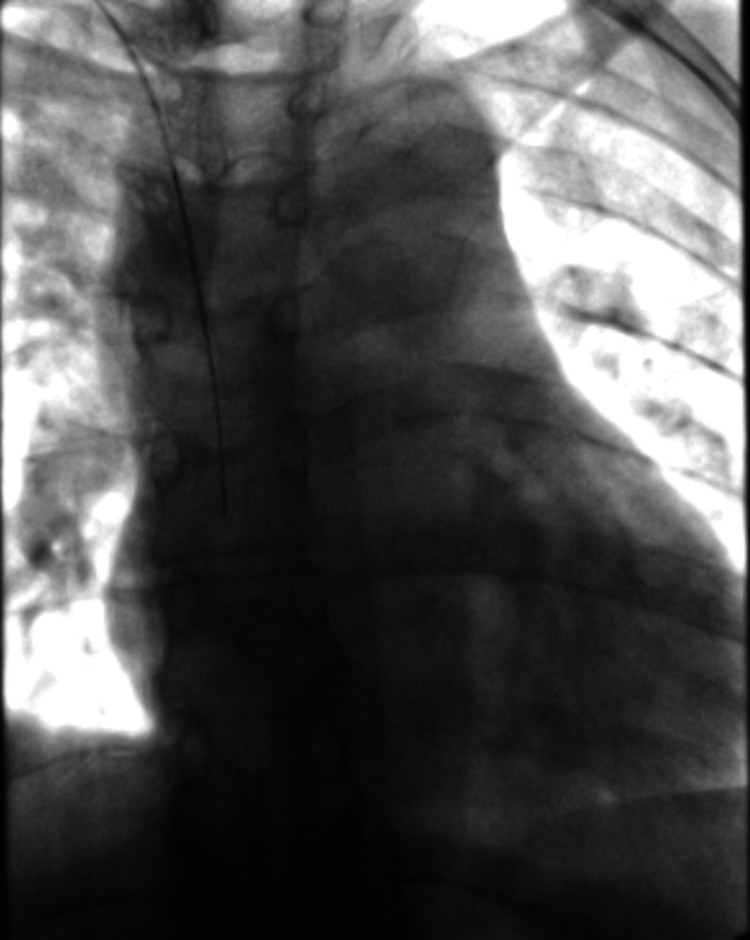
Correct positioning of the guidewire within the cavoatrial junction Correct positioning of the guidewire within the cavoatrial junction, under fluoroscopic control.

**Figure 4:**
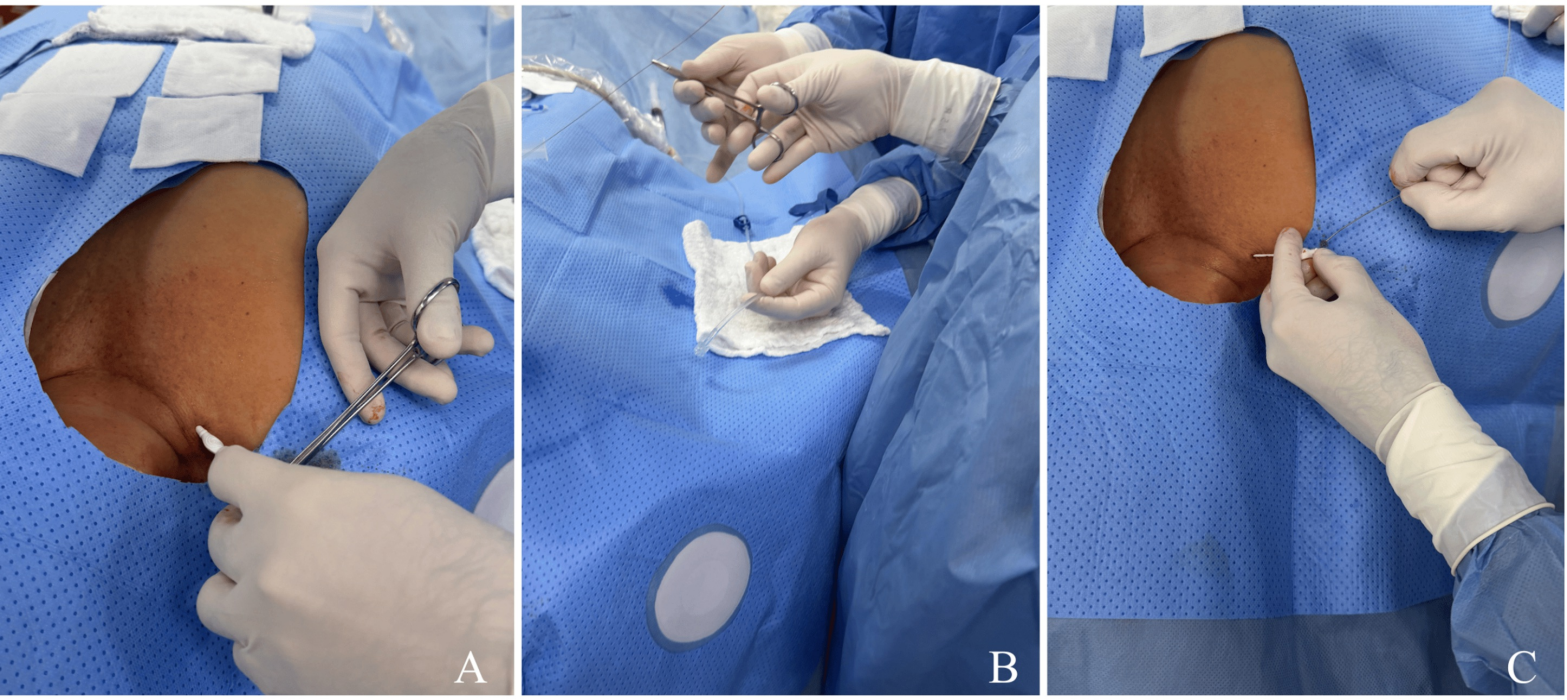
Determination of catheter length and air embolism prevention maneuver (A, B) The guidewire was marked by placing a hemostat clip at its proximal end using a needle holder to determine the appropriate catheter length. (C) The introducer needle and dilator were subsequently removed, and a rapid occlusive maneuver was performed to prevent air embolism.

Before the puncture, a 3 mm incision was made in the subcutaneous tissue, and blunt dissection of the soft tissues was performed using mosquito forceps. The puncture was then performed under color Doppler ultrasound guidance at the supraclavicular level, near the jugulosubclavian junction, using a micropuncture set (Merit MAK™ Mini Access Kit, Merit Medical, South Jordan, UT). The guidewire from the micropuncture set was advanced, and the introducer needle and dilator were subsequently removed (Figure 2). Under fluoroscopic control, the correct positioning of the guidewire within the cavoatrial junction was confirmed (Figure 3). The guidewire was then marked by placing a hemostat clip at its proximal end with a needle holder to determine the appropriate catheter length (Figure 4).

The puncture of the anterior wall of the internal jugular vein was performed as close as possible to the jugulosubclavian junction to prevent catheter kinking, using the micropuncture set. Through this access, 20 mL of non-ionic iodinated contrast medium was injected for venocavography, confirming normal patency and caliber of the jugular, brachiocephalic, and superior vena cava, with unobstructed contrast flow into the right atrium. No evidence of stenosis, membranous obstruction, anatomic variant, or aneurysmal dilatation contraindicating the procedure was observed.

Pocket Creation

**Figure 5:**
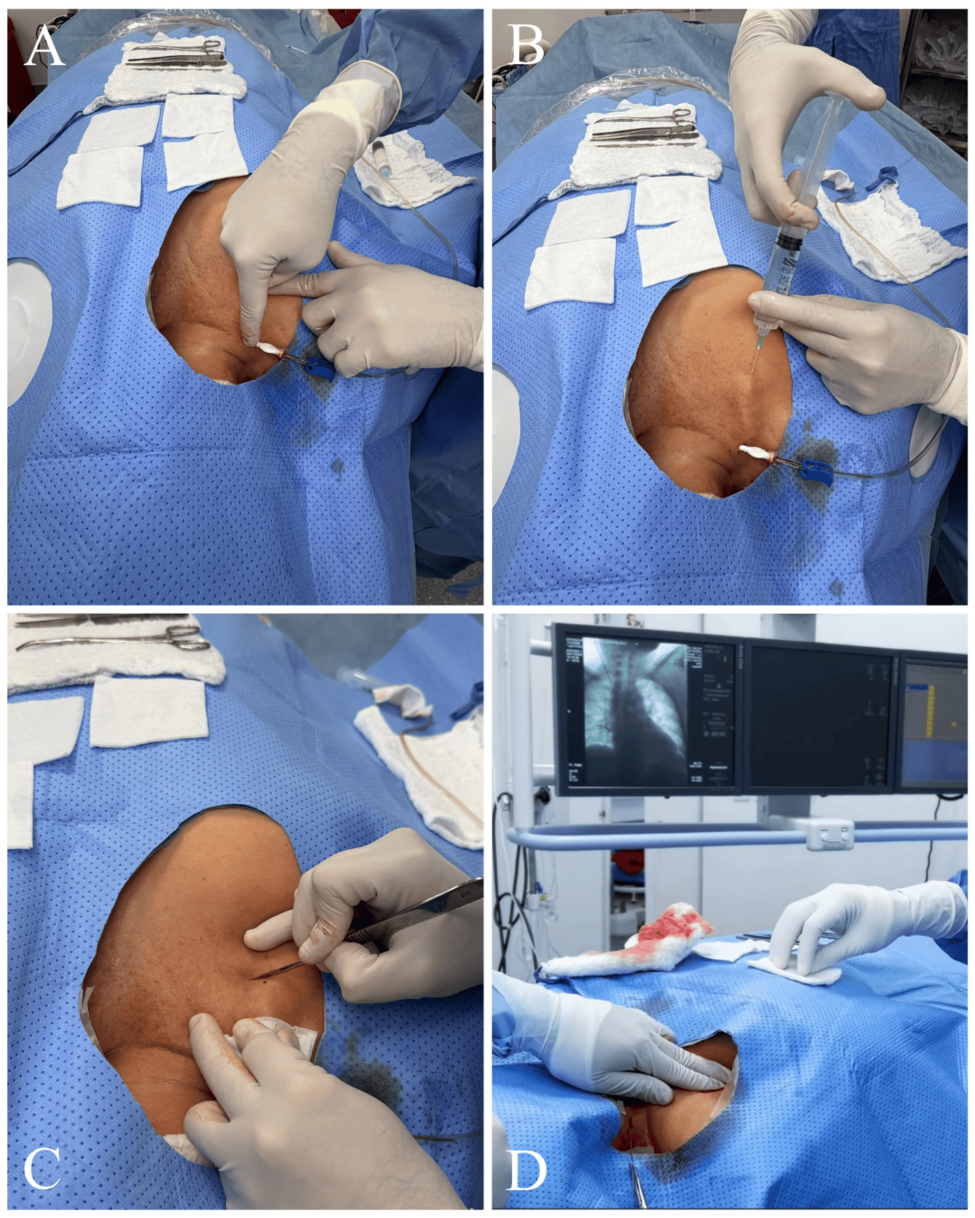
Creation of the subcutaneous pocket and implantation of an implantable venous access port (A) Gentle blunt dissection using the operator’s little finger. (B) The pocket was irrigated with sterile saline solution mixed with gentamicin. (C, D) A totally implantable venous access port was implanted.

**Figure 6:**
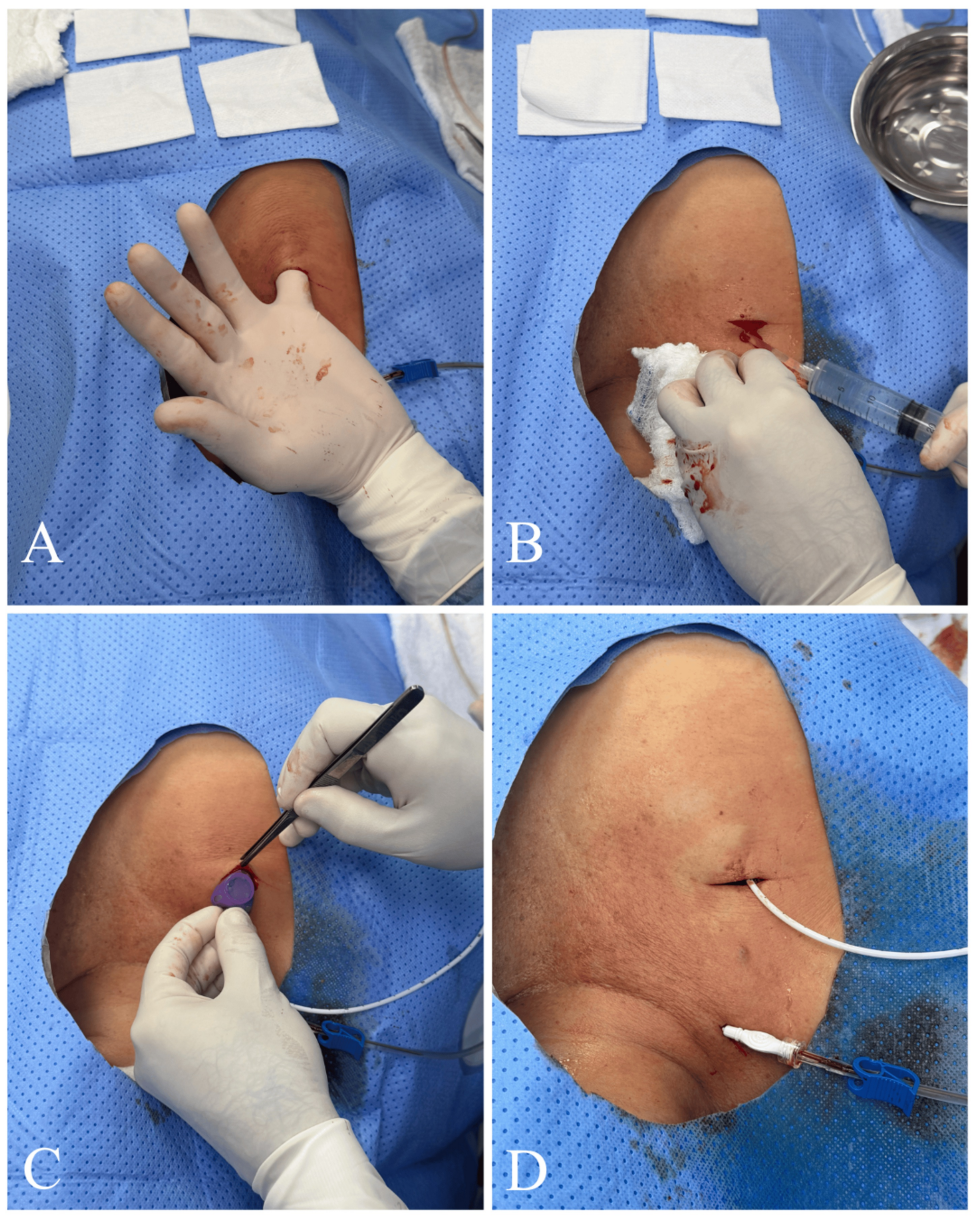
Creation of the subcutaneous pocket and implantation of an implantable venous access port (A) Gentle blunt dissection, preferably using the operator’s little finger. (B) The pocket was irrigated with sterile saline solution mixed with gentamicin. (C, D) A totally implantable venous access port was implanted for chemotherapy administration.

Subsequently, under local anesthesia with 15 mL of 2% lidocaine without epinephrine, a prepectoral subcutaneous pocket was created (Figure 5). Gentle blunt dissection was performed, ideally using the operator’s little finger (Figure 6). If bleeding occurred (which was rare), hemostasis was achieved with local pressure, and only in exceptional cases with electrocautery (Figure 5). The pocket was irrigated with 100 mL of sterile saline solution mixed with two 80 mg of gentamicin (Figure 6).

A PowerPort® Implanted Port System (Becton Dickinson, Franklin Lakes, NJ), 8 Fr, was then implanted for chemotherapy administration and secured to the subcutaneous tissue with three reverse sutures: one on the right lateral side of the chamber, one on the left lateral side, and one medially to the device, using 3-0 polyglactin 910 sutures (Vicryl®, Ethicon, Somerville, NJ) (Figure 6). This technique minimizes dehiscence and ensures a firm fixation of the chamber within a snug pocket.

Tunneling and Catheter Placement

The chemoport tunneler was then advanced through the subcutaneous tissue from the center of the incision toward the supraclavicular puncture site. The tunneler was advanced smoothly through the subcutaneous layer, avoiding the dermis and epidermis to prevent catheter exposure through the skin.

**Figure 7:**
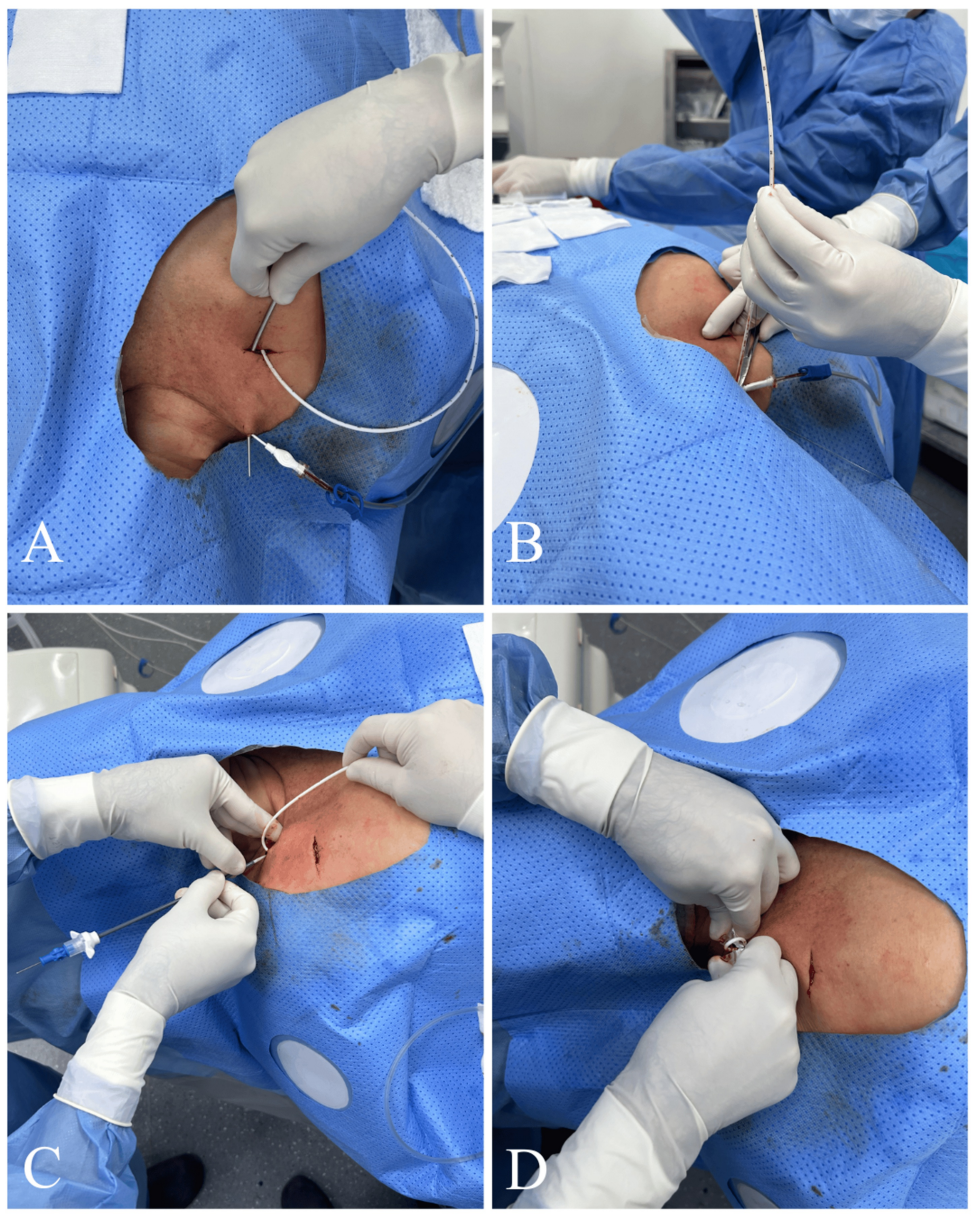
Tunneling and catheter placement (A) The chemoport tunneler was advanced through the subcutaneous tissue from the center of the incision toward the supraclavicular puncture site. (B) The catheter was cut according to the previously determined fluoroscopic measurement, subtracting 3-4 cm from the original length. (C) The peel-away introducer was advanced over the micropuncture guidewire. (D) The peel-away sheath was subsequently removed.

**Figure 8:**
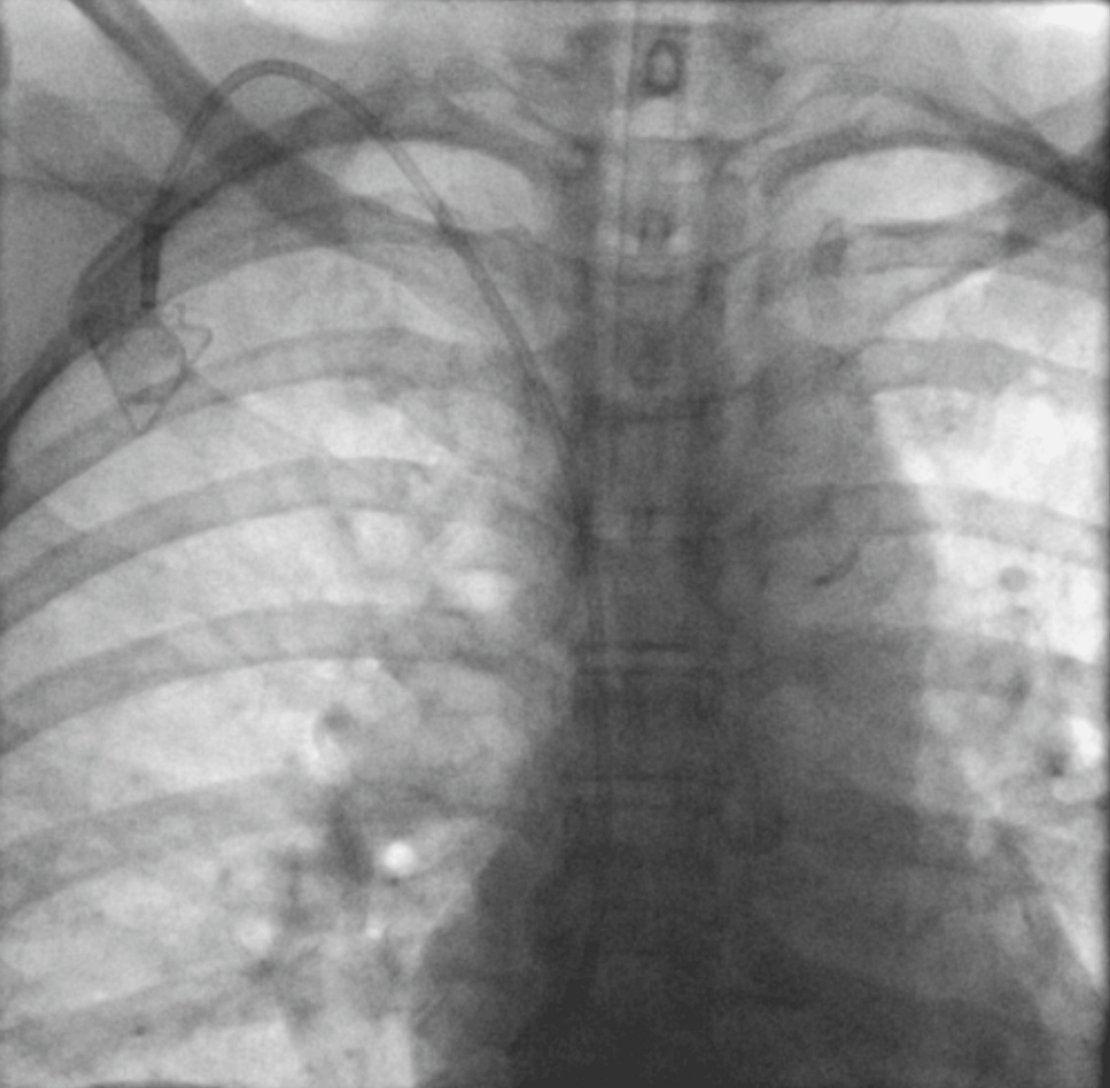
Right internal jugular vein insertion Right-sided implantable port catheter, with the catheter tip positioned superficially within the right atrium. Anteroposterior chest radiograph obtained with a C-arm demonstrating correct catheter placement at the cavoatrial junction.

The catheter was then connected and passed through the subcutaneous tunnel, emerging at the puncture site. It was cut according to the previously determined fluoroscopic measurement, subtracting 3-4 cm from the original length. The peel-away introducer was advanced over the micropuncture guidewire, occasionally under fluoroscopic guidance when resistance was encountered, to prevent complications such as superior vena cava rupture (Figure 7). The catheter was then introduced through the peel-away sheath. Correct positioning of both the catheter tip located at the cavoatrial junction or within the cavoatrial junction or within the superficial sector of the right atrium, and the port chamber was confirmed fluoroscopically using a C-arm unit (Figure 8).

Closure and Post-procedural Care

**Figure 9:**
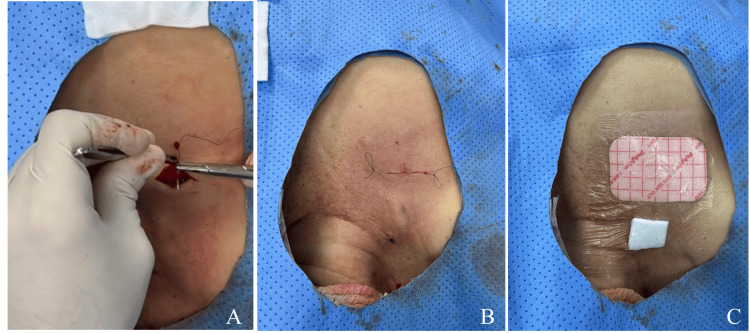
Wound closure and dressing (A, B) The incision was closed using an intradermal suture. (C) The wound was covered with a nonadherent dressing and sterile gauze.

The port chamber was flushed with sterile saline solution at the end of the procedure. The incision was closed with an intradermal 3-0 silk suture to be removed after eight days (Figure 9). Immediate procedural complications, radiation dose, contrast volume, and total fluoroscopy time were recorded.

Patient education was provided regarding warning signs and pharmacologic Management, which included nonsteroidal anti-inflammatory drugs (NSAIDs) and, in some cases, acetaminophen 1 g every six hours, as well as cephalexin 500 mg every six hours for five days. Catheter use was recommended 24 hours after insertion. Video footage of the described technique is available in the supplementary material.

## Results

During the study period, 160 patients underwent a total of 170 procedures with the interventional radiology unit: 155 (91.17%) for chemoport insertion and 15 (8.82%) for device removal. All patients had an oncologic diagnosis. Among these 160 patients, there were a total of 94 (58%) females and 66 (41.25%) males in the study group.

**Figure 10:**
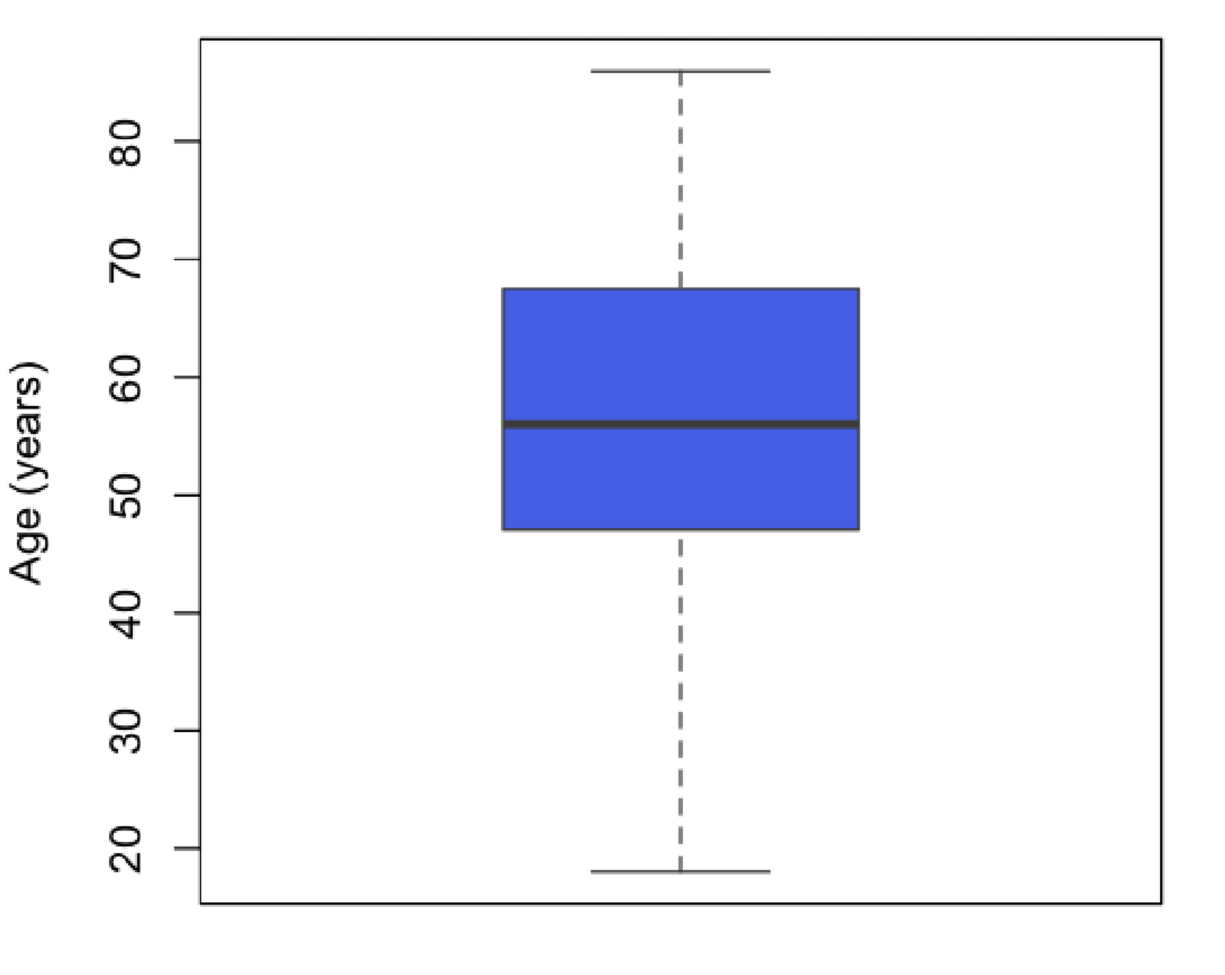
Age distribution of the study population Box plot illustrating the distribution of patient age in years. The box represents the interquartile range, the horizontal line within the box indicates the median, and the whiskers denote the minimum and maximum values.

Among patients undergoing chemoport insertions, 88 (56.77%) were female, and 67 (43.22%) were male. The mean age was 55.71 years (IQR 47.0-67.5). Age showed a non-normal distribution (Shapiro-Wilk W = 0.97, p = 0.002) (Figure 10). Overall, the three most recorded cancers were gastrointestinal in 79 (48%), breast in 43 (27%), and hematological in 10 (6%) cases (Table 1).

**Table 1:** Distribution of cancer types among the study population The table summarizes the frequency and percentage of cancer types recorded based on the total of patients (n = 160). Data are presented as absolute numbers (n) and percentages (%).

Cancer type	n	%
Gastrointestinal	79	49.38%
Breast	43	26.88%
Hematological	10	6.25%
Gynecological	9	5.63%
Head and neck	5	3.13%
Testicular	5	3.13%
Others	4	2.50%
Oral	2	1.25%
Renal	2	1.25%
Pulmonary	1	0.63%
Total	160	100.00%

The total number of recorded cancers was analyzed after accounting for potential duplicates through patient identification numbers, resulting in a total of 160 unique patients. Among female patients, the most prevalent malignancy was breast cancer in 42 (44.68%) cases, followed by gastrointestinal in 37 (39.36%) cases and gynecological in nine (9.6%) cases. In contrast, among male patients, the most frequent malignancy was gastrointestinal in 42 (63.64%), followed by hematological in seven (10.61%) cases and head and neck in five (7.58%) (Tables 2-3).

**Table 2:** Distribution of cancer types among female patients The table presents the frequency and percentage of cancer types recorded among female patients based on the total of patients (n = 160). Data are expressed as absolute numbers (n) and percentages (%).

Cancer type	n	%
Breast	42	44.68%
Gastrointestinal	37	39.36%
Gynecological	9	9.57%
Hematological	3	3.19%
Other	2	2.13%
Oral	1	1.06%
Total	94	100.00%

**Table 3:** Distribution of cancer types among male patients The table presents the frequency and percentage of cancer types recorded among male patients based on the total of patients (n = 160). Data are expressed as absolute numbers (n) and percentages (%).

Cancer type	n	%
Gastrointestinal	42	63.64%
Hematological	7	10.61%
Head and neck	5	7.58%
Testicular	5	7.58%
Other	2	3.03%
Renal	2	3.03%
Breast	1	1.52%
Oral	1	1.52%
Pulmonary	1	1.52%
Total	66	100.00%

**Figure 11:**
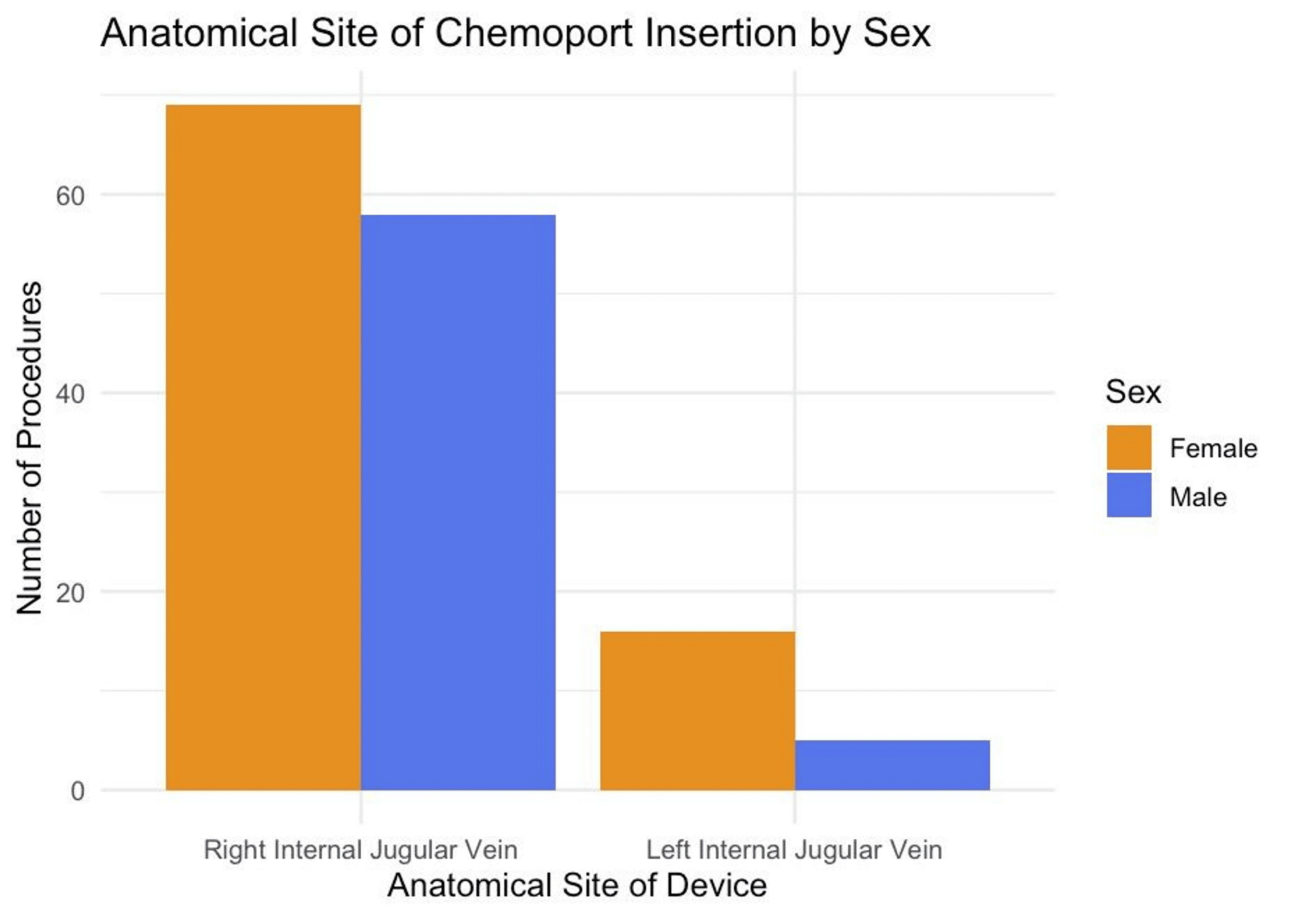
Anatomical site of chemoport insertion stratified by sex Bar chart illustrating the distribution of chemoport insertion sites according to sex, comparing the right and left internal jugular veins based on the total of chemoport insertions (n = 155).

Regarding the anatomical site of insertion, 134 (78.82%) of the chemoports were placed in the right internal jugular vein and 21 (12.35%) in the left internal jugular vein. The distribution of chemoport insertion sites stratified by sex is demonstrated in Figure 11.

**Figure 12:**
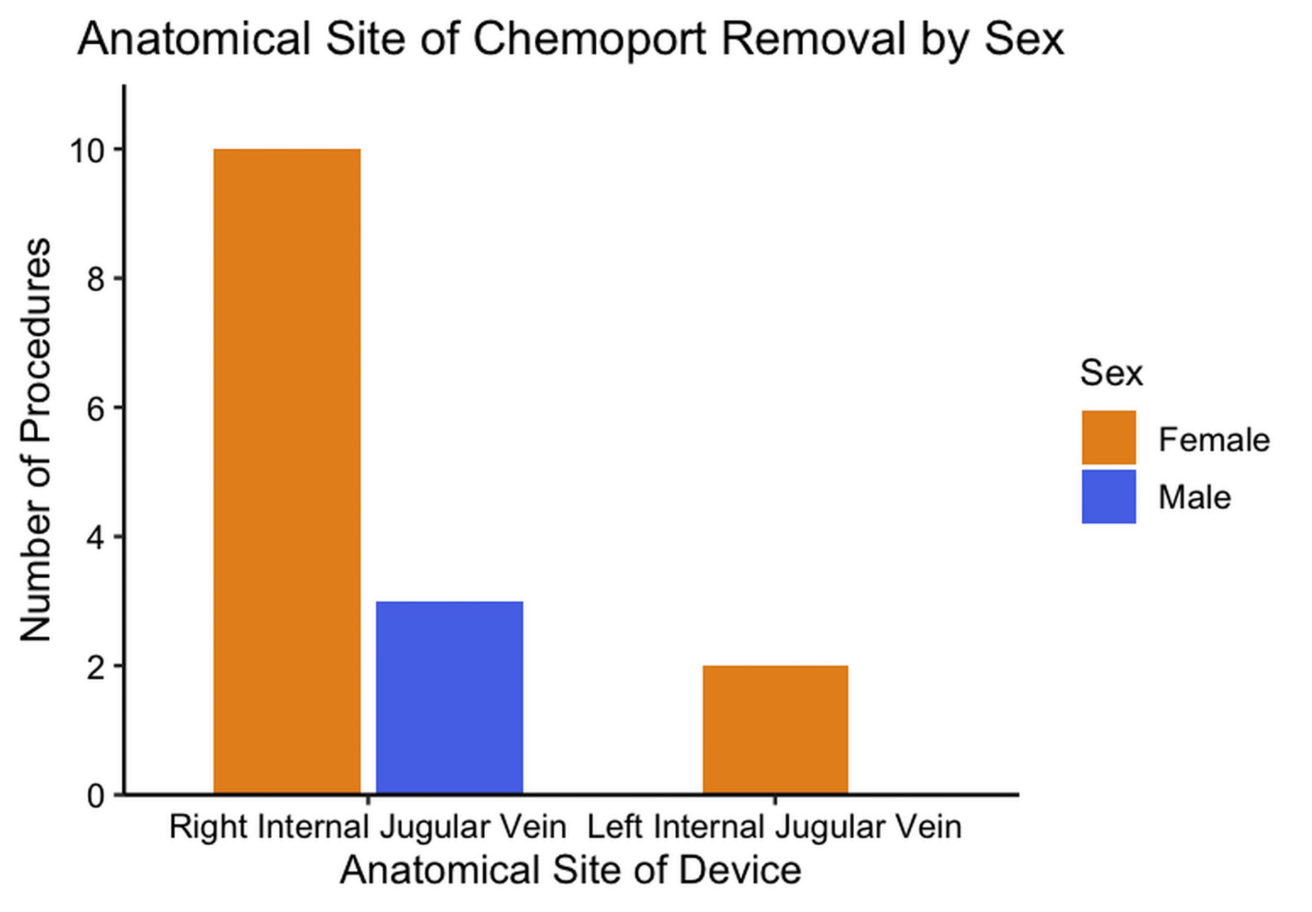
Distribution of chemoport removal sites stratified by sex Bar chart illustrating the distribution of chemoport removal sites according to sex, comparing the right and left internal jugular veins.  Based on the total of chemoport removals (n = 15).

**Figure 13:**
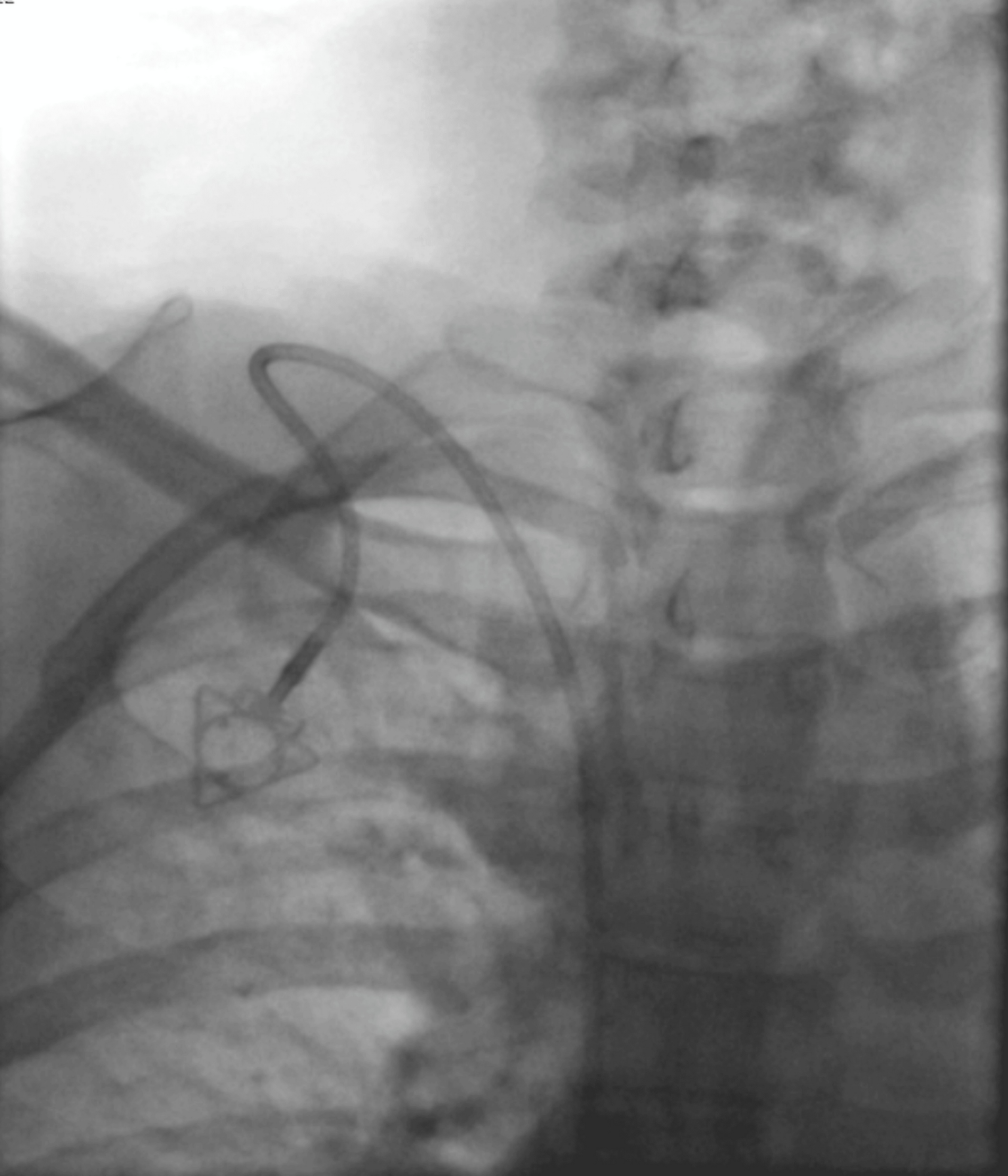
Kinked right catheter Anteroposterior chest radiograph obtained with a C-arm demonstrates a kinked right-sided catheter. This finding may be a consequence of inadequate tunneling. The patient experienced discomfort, and catheter removal was required as a result.

Finally, 15 patients underwent removal of the chemoport device; 12 (80%) were female, and three (20%) were male (Figure 12). The most common indication for device removal was the completion of cancer treatment, with a total of 10 (73.33%) cases, while complications from our chemoport insertions accounted for four (26.66%) cases among the removals. There was only one case that needed removal due to kinking of the chemoport device that was not from our insertions (Figure 13).

By anatomical site, two (13.33%) of the chemoports removed due to complications were in the right internal jugular vein, and two (13.33%) in the left jugular vein as well. The 10 removals due to treatment completion were located in the right internal jugular vein.

Regarding complications from our insertions, there were no early complications during the study period, whereas four late complications were recorded. One (25%) was discomfort, one (25%) was vena cava occlusion, and two (50%) cases were thrombosis. No other complications, such as arterial perforation, pneumothorax, infection, air embolism, pulmonary embolism, or catheter fracture or migration, were registered during the study period.

Statistical Analysis

Categorical variables were compared using Fisher's exact test, as appropriate, due to the limited number of patients used. Continuous variables were assessed for normality using the Shapiro-Wilk test and compared using the Mann-Whitney U test due to non-normal distribution. 

There was no statistically significant association between sex and implantation complications (Fisher’s exact test, p = 0.435). Similarly, the anatomical site of insertion was not associated with late complications (Fisher’s exact test, p = 0.089). Age was not statistically associated with late complications (Mann-Whitney U test, p = 0.294) (Table 4).

**Table 4:** Association between patient characteristics and complications Associations between patient characteristics and complications. Fisher’s exact test was used for categorical variables, reporting odds ratios as the test statistic. Age was compared using the Mann-Whitney U test, reporting the W statistic. ^*^IQR: interquantile range. The IQR was calculated based on age. ^†^Odds ratio could not be reliably estimated due to zero events in one comparison group.

Variable	Complication present (N)	Complication absent (N)	Test	Test statistic	p
Sex and procedural complications	1	169	Fisher’s exact	Odds ratio = Inf^†^	0.435
Anatomical site of insertion and Late complications	4	166	Fisher’s exact	Odds ratio = 6	0.089
Age and late complications	Median 42.0; IQR^*^ = (40.5-49.5)	Median 56.0; IQR^*^ = (46-67)	Mann-Whitney U	W = 432	0.294

## Discussion

Central venous port systems have been widely demonstrated to be reliable for patients requiring long-term intravenous therapies and to improve their quality of life [9]. Our results and experience with chemoport devices support this finding, confirming that this procedure can be safely performed by interventional radiologists under a methodologically consistent approach. This type of procedure can be performed using different techniques, including percutaneous insertion guided by anatomical markers, under ultrasound guidance, or surgical access to the cephalic vein. However, performing this procedure by interventional radiology offers a safe and reliable option to the oncological patient [10].

Complications rated for this procedure by surgeons and interventional radiology vary from 17.85 to 19%, respectively [11,12]. Performing the procedure by interventional radiology, along with an adequately designed procedure, may help reduce the rates of complications, as there were no early complications, and only four patients developed late complications. Two of the complications occurred in the right internal jugular vein; this is consistent with the fact that most catheter insertions are generally made in the right internal jugular vein: additionally two occurred in the left internal jugular vein, the laterality of this vein has been associated with higher rates of thrombotic and infectious complications due to a longer intravascular course and sharper angulation into the brachiocephalic vein [12].

According to the literature, the most frequent early complications are inadequate catheter positioning, arterial perforation and bleeding, pneumothorax, air embolism, and cardiac dysrhythmias [9]. None of these complications were observed during our study period from our insertion cohort, highlighting the safety and efficiency of the described technique [9]. Late complications most commonly include infection, venous thrombosis, pulmonary embolism, venous stenosis, catheter migration, and fracture [9]. In our study, only one case of thrombosis and discomfort was registered, further supporting the safety and reliability of the procedure when performed by the interventional radiology team.

Another important aspect to consider is the limited availability of resources in the country in which this study was conducted. Many patients have limited access to health services, making the availability of safe and reliable procedures critical to reduce emergency room visits and hospitalizations. In this context, the implementation of safe, efficient, and reproducible techniques becomes crucial. Other local primary studies have documented low complication rates (8.5%), but higher than in our study, among patients undergoing implantable central venous catheter placement for chemotherapy performed by interventional radiologists in the Central Military Hospital [8]. Comparable studies from high-income countries have reported similar or slightly higher complication rates, suggesting that procedural safety can be maintained even in resource-limited settings when standardized techniques are applied [3,6,7]. These findings reinforce the efficacy and practicality of this procedure in the Colombian context and its generalizability in resource-limited countries.

Our technique offers several advantages. It is performed under strict aseptic and antiseptic conditions using chlorhexidine, which contributes to a significant reduction in infection rates, as in our sample, where the incidence of infection was 0%. In addition, the procedure allows verification of the anatomy through Doppler ultrasound and fluoroscopic guidance, ensuring proper placement of the catheter and monitoring in real time to prevent kinking.

Another advantage is that the technique only needs a minimal incision, which contributes to maintaining adequate hemostasis; the pocket is manually created as small as possible, allowing the port chamber to be inserted under pressure and correctly positioned. This approach prevents the port and the catheter from migrating, maintaining their stability and functionality.

Finally, the low rate of complications observed in our population highlights the quality and safety of our technique. Further evaluation and broader implementation of this type of procedure for other patient groups, such as those needing parenteral nutrition, may represent a safe and effective option within the resource-limited healthcare system in Colombia and other similar countries. The retrospective nature of this study may limit the capacity to identify complications, as minor short-term complications may be underestimated.

Regarding limitations, this study may be subject to selection bias, as it relied on retrospective review of electronic medical records and excluded cases with incomplete documentation. Additionally, due to the retrospective design, information bias may have occurred as a result of variability in data completeness and accuracy of recorded clinical information. The single-center design and the low number of observed complications limit the study’s statistical power to detect significant associations and restrict the generalizability of the findings. Although efforts were made during data collection to minimize these biases, the retrospective nature of the study inherently limited the authors’ ability to fully address them. Despite these limitations, this study provides valuable real-world data from a public hospital setting in Colombia.

## Conclusions

Chemoport insertion using ultrasound-guided internal jugular vein access with fluoroscopic confirmation is currently considered the preferred approach, as it enhances procedural success and reduces complication rates. The findings of this study suggest that this technique is safe when performed by interventional radiologists. Within the resource-limited healthcare context of Colombia, the procedure is efficient, with most patients not requiring prolonged hospitalization or intensive post-procedural follow-up. However, safety-related conclusions are limited by the retrospective design, which restricts the precision of risk estimates and limits comparative inference. Further research is warranted to evaluate its effectiveness in other chemoport indications, particularly among patients requiring long-term intravenous therapies such as parenteral nutrition, within Colombia and across Latin America.
